# 

**Published:** 1908-07-11

**Authors:** 


### CONTENTS.

| | PAGE |
|---|---|
| **Artificial Respiration** | 379 |
| **Doctors and Socialism** | 380 |
| **Medical Opinion and Movement** | 382 |
| **Hospital Clinics—** | |
| Some Common Diseases of the Eye: their Diagnosis and Treatment. By Percy Dunn, F.R.C.S. | 385 |
| Consultations | 388 |
| **Medicine—** | |
| Secondary Deposits in Left Supra-Clavicular Glands in Cases of Malignant Disease of Abdominal Organs | 389 |
| Fat Absorption after Gastrojejunostomy | 390 |
| **Surgery—** | |
| Acute Retention of Urine - II. | 391 |
| Some Aspects of Intestinal Obstruction | 392 |
| **Obstetrics—** | |
| The Treatment of Labour Complicated by Fibroid Tumours of the Uterus | 393 |
| **Anæsthetics—** | |
| Lung Troubles After Anæsthetics | 394 |
| **Public Health and Hygiene—** | |
| The Arbitrament of the Magistrate | 395 |
| **Laryngology and Rhinology—** | |
| Nasal Obstruction—II. | 396 |
| **The Royal Army Medical Corps Section—** | |
| The British Red Cross Society and the Territorial General Hospitals | 397 |
| **Practical Notes on Diagnosis and Treatment** | 398 |
| **Hospital Administration—** | |
| Graduate Study on the Continent | 399 |
| **Nursing Administration—** | |
| Some Laundry Statistics | 403 |
| **Editor's Letter-Box** | 404 |

